# An Rcs Stress-Based High-Throughput Screen Reveals Novel Gyrase Inhibitors as Indirect Inducers of Cell Envelope Stress in Gram-Negative Bacteria

**DOI:** 10.1021/acsinfecdis.5c00445

**Published:** 2025-08-08

**Authors:** Laurence Cleenewerk, Alexandra Otto, Willemijn Wouters, Joost Willemse, Meiling Gao, Vladyslav Lysenko, Jeroen M. Punt, Mario van der Stelt, Nathaniel I. Martin, Peter van Ulsen, Joen Luirink

**Affiliations:** † Department of Molecular Microbiology, A-LIFE, AIMMS, 1190 VU Amsterdam, De Boelelaan 1108, 1081 HZ Amsterdam, The Netherlands; ‡ 573873 Pivot Park Screening Centre, Kloosterstraat 9, 5349 AB Oss, The Netherlands; § Institute of Biology, Microscopy Unit, 4496 Leiden University, Sylviusweg 72, 2333 BE Leiden, The Netherlands; ∥ Biological Chemistry Group, Institute of Biology, 4496 Leiden University, Sylviusweg 72, 2333 BE Leiden, The Netherlands; ⊥ Department of Molecular Physiology, Leiden Institute of Chemistry, 4496 Leiden University, Einsteinweg 55, 2333 CC Leiden, The Netherlands

**Keywords:** cell envelope stress, high-throughput screen, quinolones, gyrase, antibiotics

## Abstract

The highly impermeable cell envelope of Gram-negative bacteria is an important hurdle to the development of novel antibacterials. However, compounds that disrupt the integrity of the cell envelope can act as potent antibiotics by directly inhibiting cell growth and viability or by enhancing the penetration of other, larger antibiotics otherwise unable to pass this barrier. To identify such novel compounds, we used the European Lead Factory compound libraries to screen >500,000 small molecules for inducing the Rcs cell envelope stress response in *Escherichia coli* . We identified a series of novel 2-quinolones and 4-quinolones that target gyrase and topoisomerase IV, suggesting unforeseen effects of such compounds on the bacterial cell envelope. Here, we show that the quinolones induce a structure-dependent profile of specific cell envelope stress responses. These response profiles were observed not only for quinolone-type but also for structurally unrelated gyrase inhibitors. Importantly, DNA damage and SOS response activation alone were insufficient to explain the high levels of cell envelope stress, underscoring gaps in our understanding of the interplay between gyrase function and maintenance of cell envelope integrity. Microscopy showed structural changes that are likely related to the observed stress. Importantly, cell elongation, associated with quinolone-induced SOS stress response, also occurred in SOS-deficient bacteria. These serendipitous findings highlight both the complexity of gyrase-associated bactericidal mechanisms and the challenges in antibiotic discovery. Nevertheless, this study supports the utility of stress-based assays as sensitive phenotypic tools for identifying new antimicrobial agents.

## Graphical abstract

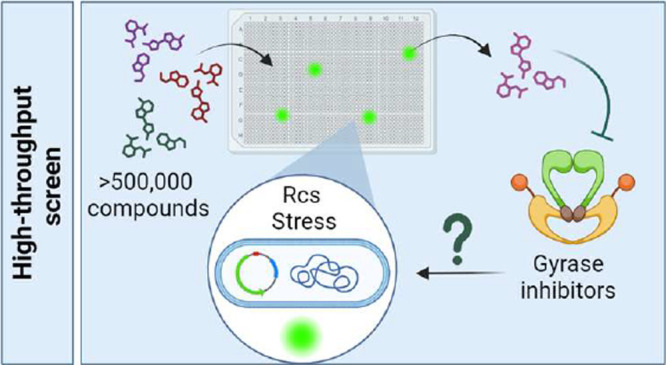

Developing antibiotics against pathogenic bacteria is of crucial importance due to increasing rates of antimicrobial resistance (AMR).^1^ Gram-negatives are of particular concern due to their intrinsic resistance to antibiotics: the structure of their cell envelope (CE) prevents most antibiotics from entering the cell and represents a major hurdle to the development of antibiotics.^2^

The CE consists of three layers: the phospholipid bilayer inner membrane (IM), the periplasm harboring the thin peptidoglycan (PG) layer, and the highly impermeable asymmetric bilayer of the outer membrane (OM). The OM contains a dense layer of anionic chains of lipopolysaccharide (LPS), which restricts the passage of large (>600 Da) and hydrophobic compounds. In addition to LPS, the OM also contains various outer membrane proteins (OMPs) that adopt a beta-barrel conformation. Some OMPs, known as porins, function as narrow channels with a cutoff of ∼600 Da to selectively allow the passage of smaller hydrophilic compounds. Additionally, multidrug efflux pumps enable the expulsion of compounds, including antibiotics, that manage to cross the OM.^3,4^

The OM is a common hurdle faced by many clinically used antibiotics that act on intracellular targets, which renders them ineffective against Gram-negatives.^3^ Recently, disrupting the OM barrier, e.g. through targeting essential processes that affect OM integrity, has emerged as a compelling approach for developing novel antibiotics against Gram-negatives, and several promising compounds have been reported.^5–7^ By directly targeting processes in the OM, compounds can circumvent both multidrug efflux pumps and the intrinsic resistance associated with the OM permeability barrier. Currently, no OM-targeting compounds apart from polymyxins are licensed for use in humans.^8^ However, recent efforts to discover such agents have been made, and several compounds acting on the essential, surface-exposed OMPs LptD and BamA have been reported.^9,10^ LptD mediates the last step in LPS biogenesis and is a target of peptidomimetic inhibitors such as murepavadin.^11^ BamA, an essential component of the beta-barrel assembly machinery (BAM), which is responsible for folding and inserting OMPs, is targeted by compounds such as darobactin and MRL-494.^12–14^ These types of compounds can have a bactericidal effect through inhibition of effective LPS and OMP transport and insertion. Inhibition of these processes has also been described to cause increased OM permeability by disrupting the essential structure of the CE. Therefore, they could be used as stand-alone antimicrobials or as potentiating agents for other antibiotics.^15^ In addition, other (intracellular) processes that are crucial for membrane integrity may also represent potential targets, such as lipoprotein biogenesis and translocation, peptidoglycan synthesis, and maintenance of the proton motive force.^16^ Overall, there are many underexplored potential targets involved in the construction and maintenance of the CE.

Identifying CE-targeting compounds remains challenging. Bacteria have evolved extensive systems that monitor and respond to CE damage, which may hamper identifying CE-disrupting compounds. In addition, bacteria may remain viable under laboratory conditions even when experiencing membrane defects. For instance, the BamA-depleted *E. coli* strain *bamA101*, which expresses as little as 10% of wild-type BamA levels, exhibits normal growth despite defects in membrane integrity.^17^ Moreover, a deacylated derivative of polymyxin B, called polymyxin B nonapeptide (PMBN), maintains its ability to permeabilize the OM of Gram-negative bacteria and sensitizes them to larger (>600 Da) antibiotics with intracellular targets, although it exhibits greatly reduced bactericidal properties on its own.^18^ Since antibiotics are classically identified using screens based on viability, a different, more sensitive approach is warranted to identify membrane-disrupting compounds that are likely detrimental in vivo.

We have recently developed phenotypic assays that report on bacterial CE stress responses induced upon membrane damage.^4,19,20^ The stress responses include sigma *E* (σE), Cpx, and Regulation of Capsular Synthesis (Rcs), with each system having distinct, but also partially overlapping triggers from various environmental changes and toxic molecules. Briefly, the σE system senses the accumulation of unfolded OMPs in the periplasm and divergent forms of LPS. The Cpx response is activated by various triggers, such as defective PG synthesis or defective protein translocation across the IM, misfolded IM or periplasmic proteins, and aberrant phospholipid composition and biogenesis.^21,22^ Lastly, the Rcs response is sensitive to the broadest range of triggers and is initiated upon defects in LPS and PG synthesis, erroneous LPS and lipoprotein trafficking, and malfunctioning of the BAM.^19^

We have previously used the σE stress reporter assay in high-throughput screens (HTS) and identified synthetic compounds that inhibit BAM-mediated OMP biogenesis.^4,19,20^ However, a major drawback of this assay was the need to overexpress a BAM substrate to increase assay sensitivity, making it technically more challenging for high-throughput screening purposes. In addition, the screens identified only a limited number of hits, thus prompting us to use the more sensitive Rcs stress reporter assay. Given the broad array of triggers that induce Rcs stress, we reasoned that employing the Rcs stress reporter in an HTS could yield a broad spectrum of potential membrane-interfering compounds, with a variety of potential targets involved in membrane processes.

Here, we used the Rcs stress reporter assay to screen >500,000 small molecules that reside in the European Lead Factory (ELF) program.^23^ While we expected the screen to result in a variety of hit compounds specific for various CE targets, we obtained a limited set of hits, the majority of which (8 out of 12) belonged to the known antibiotic class of quinolones. This type of compound inhibits gyrase and/or topoisomerase IV, two type II topoisomerases located in the cytosol, where they exert crucial functions during DNA replication.^24^

Surprisingly, the quinolones were shown to be potent inducers of the Rcs but also the σE and Cpx stress responses. These responses seemed to be an indirect effect secondary to gyrase inhibition, rather than being specific to quinolones. The nature and kinetics of the observed stress responses were dependent on the specific inhibitor structure and may be related to the type of DNA damage induced. Morphological studies using Scanning Electron Microscopy (SEM) and fluorescence microscopy showed drastic changes in CE structure upon gyrase inhibition. Importantly, DNA damage and activation of the SOS response appeared not solely responsible for CE stress. Altogether, the serendipitous identification of gyrase inhibitors during our screen aimed at finding CE stress-inducing compounds highlights both the incomplete understanding of the bactericidal consequences of gyrase inhibition and the challenges in identifying novel classes of antibiotics. Overall, the findings support the usefulness of stress-based assays as a sensitive and selective tool for the identification of antimicrobial compounds.

## Results

### Fluorescence-Based High-Throughput Screen (HTS) Identifies Quinolones as Inducers of Rcs Cell Envelope Stress

We adapted the reporter assay based on the Rcs stress system for HTS in a screening program at the ELF as the first bacterial phenotypic screen used in this setting.^4,23,25^ The assay was performed in a 1536-well format using 25 μM PMBN as a reference (set at 100% effect). At this concentration, PMBN disrupts the OM without being bactericidal and induced a robust Rcs stress response that resulted in high and reproducible fluorescence values.^19^

The ELF screened their extensive and diverse library consisting of >500,000 small molecules at a concentration of 10 μM. The assay window (S/B) and Z’ values remained stable (2.0–15.2 and 0.50–0.90, respectively) throughout the screening campaign. The detailed standard screening procedure of the ELF is outlined elsewhere.^26^ An overview of the selection process of the screening campaign performed here is outlined in Figure 1. Initially, 340 compounds with an effect ≥20% of the reference compound PMBN were considered biologically active. Usually, up to 1% of the input library is selected for confirmatory, dose–response, orthogonal and deselection assays. Because the number of biological actives identified in this assay was well below the 1% limit, no orthogonal assays were performed. Instead, all compounds were reordered and tested as dilution series in the Rcs assay to generate dose–response curves (DRCs). Each compound’s pEC50 (−log of EC50, defined as the concentration (mol/L) at which 50% effect is achieved) was determined, and compounds with a pEC50 ≥ 4.7 were selected for further analysis. Next, the compounds were subjected to a mammalian cell-based cytotoxicity assay to exclude toxic compounds that may have a generic effect on membrane integrity. Compounds that reduced mammalian cell viability by 50% were considered cytotoxic. The qualified hit list (QHL) was then generated following triaging based on LC-MS analysis and pEC50 values.

**Figure 1:**
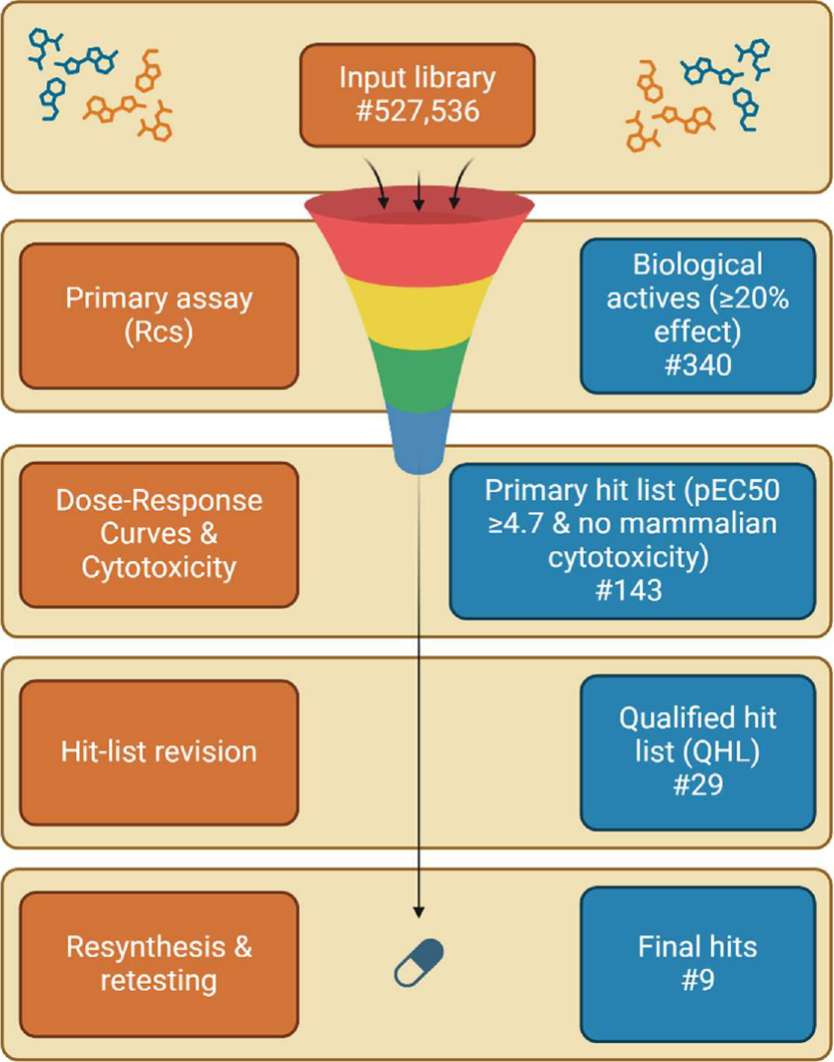
Screening workflow leading to the final hits. Compounds with >20% effect were considered biologically active. These compounds were reordered and retested in the primary assay (Rcs) in a concentration range to determine pEC50 (−log of concentration (mol/L) at which 50% effect is observed) values. Compounds with a pEC50 ≥ 4.7 that did not show mammalian cytotoxicity (<50% cell death) in the deselection assay form the primary hit list. Following hit-list revision, 29 compounds were selected for the qualified hit list (QHL), of which 9 compounds were successfully resynthesized and shown to retain activity.

The QHL consisted of 29 compounds, the majority of which (22) were quinolones, sharing a scaffold known for antibiotic compounds that interfere with gyrase/topoisomerase IV activity.^27^ The remaining 7 compounds were singletons. Based on criteria such as activity, toxicity, drug-likeness and structural novelty, the 29 compounds were further triaged, after which the 12 most interesting compounds were selected for resynthesis. One singleton could not be resynthesized and was not considered further. Next, the remaining 11 compounds were retested as dilution series in the Rcs assay to generate DRCs and confirm their pEC50 values. Two resynthesized singletons showed no activity upon retesting and were discarded. The remaining 9 compounds exhibited consistent pEC50 values and were used for further analysis (Table S1).

The final selected hits comprised four 4-quinolones, three 2-quinolones, and two singletons. An overview of these compounds is shown in Table 1. The quinolones were grouped into clusters based on their structure: Cluster A (CluA) represents the 4-quinolones and Cluster B (CluB) the aminopiperidine-linked 2-quinolones. The CluA compounds were derivatives or analogues of existing fluoroquinolone (FQ) antibiotics. The CluB compounds shared similarities with a class of aminopiperidine-linked compounds being investigated as novel bacterial topoisomerase inhibitors (NBTIs).^28^ Remarkably, the GSK compound gepotidacin, which has recently been approved by the FDA for use in uncomplicated urinary tract infections, shares many structural features with compound **B1.** ^29,30^

**Table 1:**
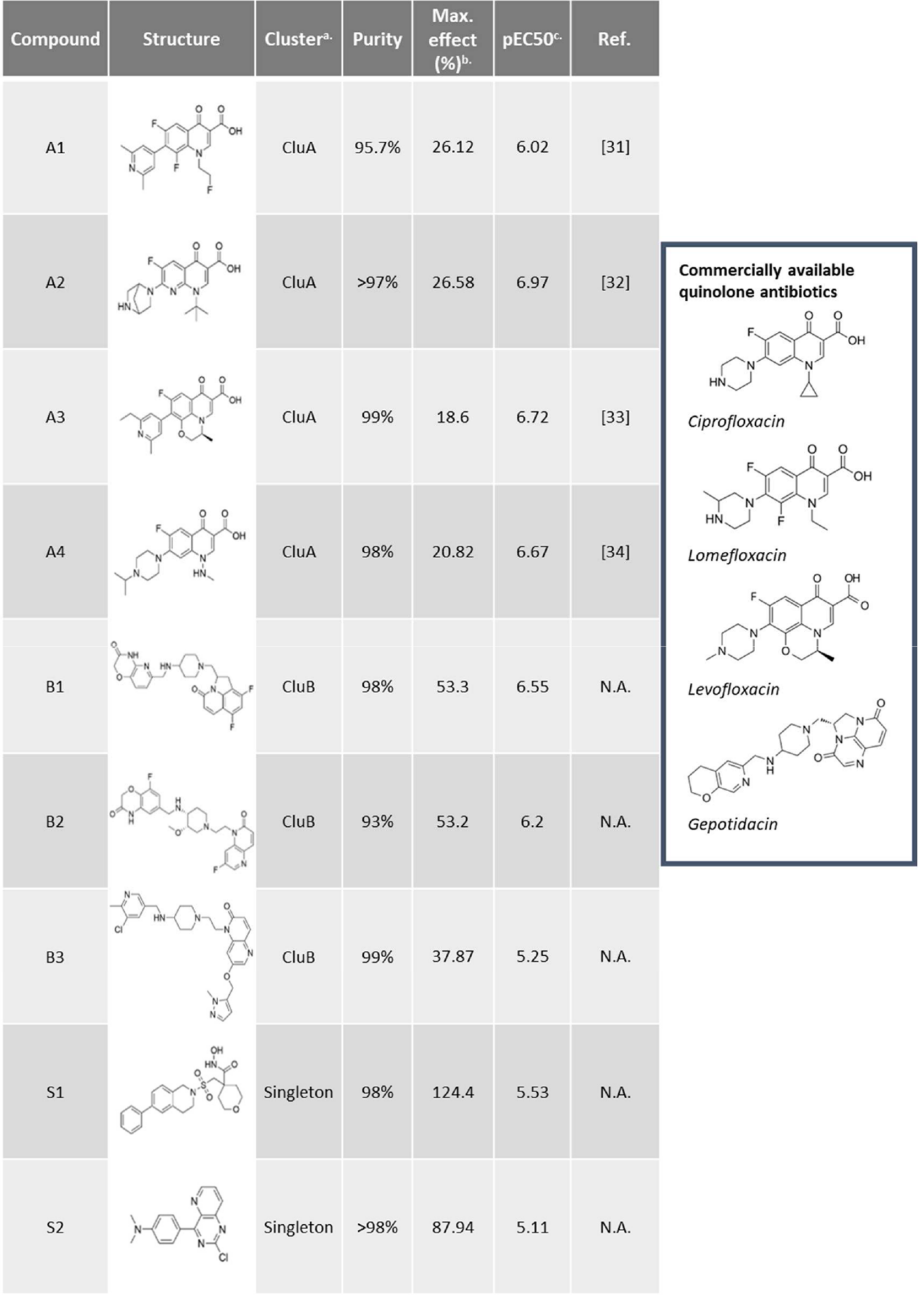
Hit Compounds of the Rcs Reporter Screen of >500,000 Small Molecules^31–34^

a Cluster A (CluA): 4-quinolones; cluster B (CluB): 2-quinolones.

b Max. effect compared to 25 μM PMBN.

c pEC50 (-log of EC50, defined as concentration (mol/L) at which 50% activity is observed) derived from dose–response curves of compounds tested in a range of 20 nM–20 μM (7 dilutions) in the Rcs stress assay.

While the assay was designed to identify compounds interfering with CE integrity, the hit list of the screen comprised largely quinolones, which likely target the intracellular gyrase/topoisomerase IV.^35,36^ Nevertheless, the hits belong to a class of compounds known for antibacterial properties, warranting further investigation into their potential as antibiotics. In addition, the screening outcome sparked our interest in the underlying mechanism of quinolone-induced CE stress. The remainder of this study examines the antibacterial properties of the hit compounds, with a focus on their CE stress-inducing characteristics.

### Confirmation of Activity of Selected Compounds under Laboratory-Scale Assay Conditions

Following the initial screen at the ELF, the compounds were further validated in our lab in a 96-well format by measuring the fluorescence intensity of compound-treated reporter cells at 10 μM compared to 25 μM PMBN (set at 100% effect) after 150 min of treatment (Figure 2). Because the screen yielded mostly quinolones, several commercial FQs were included in the assay to study their stress-inducing potential at the same concentration. MRL-494, a known OM-disrupting compound and inhibitor of BamA that induces Rcs stress, was also included.^14^ In addition, chloramphenicol was included at 10 μM as a non-stress-inducing control antibiotic. All resynthesized ELF compounds induced fluorescence with intensities similar to those found during the screening campaign, except for singleton **S2**, which showed high autofluorescence. Because this compound also showed a propensity to aggregate, it was not investigated further. Singleton **S1** emerged as a particularly strong inducer of Rcs stress, reaching 96.8% effect, similar to MRL-494 (100.4%). Since **S1** is structurally distinct from the quinolones, it will be addressed separately in future studies. CluB compounds induced fluorescence with 36.3–50.4% effect compared to 25 μM PMBN, whereas CluA compounds reached 14.8–22.0% effect. These responses were comparable to the values obtained during HTS campaign at the ELF. The FQs induced fluorescence between 3.5 and 16.0% at this concentration compared to untreated control cells, probably due to their strong antibacterial properties, causing cell death at the tested concentration.

**Figure 2:**
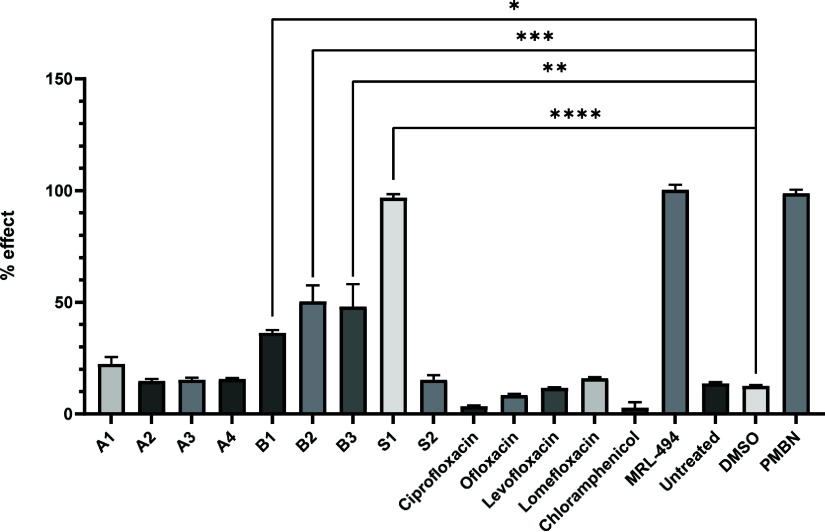
Compound-mediated levels of Rcs stress-induced fluorescence. *E. coli* TOP10F’ cells harboring a plasmid expressing mNG under control of the *P*_rprA_ promoter were treated with 10 μM of the ELF hit compounds, 10 μM FQs (ciprofloxacin, ofloxacin, levofloxacin, lomefloxacin), 25 μM PMBN, 50 μM MRL-494 or 10 μM chloramphenicol. Fluorescence was measured immediately after adding compounds (*T* = 0) and after 150 min (*T* = 150). Fluorescence at *T* = 0 was subtracted from fluorescence at *T* = 150. Values are expressed as the % effect compared to fluorescence induced by 25 μM PMBN. Error bars represent standard deviation of two independent experiments performed in triplicate. Data were analyzed using a one-way ANOVA with Dunnett’s multiple comparisons. *: *p* < 0.05; **: *p* < 0.005 ***: *p* < 0.001 ****: *p* < 0.0001.

Overall, these findings confirm all hits from the screen except one as inducers of the Rcs stress response. The remainder of this study will focus on the relationship between the quinolones and CE stress.

### Identified Quinolones Are Active against Gram-Negative and -Positive Species

To assess the antibacterial properties of the quinolone hits, the minimal inhibitory concentration (MIC) was determined. The selected panel included clinical isolates from the ATCC collection,^37^ as well as laboratory strains of *E. coli* . The MIC values are shown in Table 2.

**Table 2:** MIC Values

		MIC (μg/mL)						
cluster	compound	E. coli 25922^a^	E. coli BW25113^a^	E. coli TOP10F’^b^	K. pneumoniae 13883^c^	P. aeruginosa 27853^c^	A. baumanni 9955^c^	S. aureus USA 300^c^
CluA	A1	0.5	0.25	<0.03	1	16	1	1
	A2	0.125	0.06	<0.03	0.125	1	0.5	8
	A3	0.125	0.125	<0.03	1	8	0.5	0.5
	A4	0.25	0.25	0.125	0.5	16	2	64
CluB	B1	0.5	0.25	<0.03	1	8	0.25	0.06
	B2	2	1	0.06	4	8	1	0.25
	B3	16	16	2	64	>64	8	0.125
Fluoro-quinolone	ciprofloxacin	0.015	0.015	<0.007	0.015	0.25	0.125	16
NBTI	gepotidacin	2	1	0.06	4	8	16	0.25

a Laboratory *E. coli* strain.

b Screening strain.

c Clinical isolate from the ATCC collection.

Overall, CluA compounds had MIC values between 0.06 and 1 μg/mL in clinical Gram-negative strains, except for **Pseudomonas aeruginosa*,* where the MIC values were higher (1–16 μg/mL). CluA compounds showed generally higher MIC values (0.5–64 μg/mL) against *Staphylococcus aureus* than Gram-negatives, apart from *P. aeruginosa* . CluB compounds showed lower MIC values against *S. aureus* than the CluA compounds, with values between 0.06 and 0.25 μg/mL, and were active against clinical Gram-negative isolates at values between 0.5 and 64 μg/mL, with the highest values for *P. aeruginosa* .

The standard-of-care FQ ciprofloxacin had the lowest MIC values against all Gram-negatives but higher MIC values (16 μg/mL) against *S. aureus* than all other quinolone compounds apart from **A4**. Gepotidacin showed comparable MIC values to compound **B2** for Gram-negatives, except against *A. baumannii* . Compounds **A2** and **B1** stood out as the most potent representatives of their respective clusters. Importantly, compound **B1** had lower MIC values than gepotidacin, showing its potential as an antibiotic candidate.

### Stress Response Signatures Are Characteristic for Specific Compound Classes

To further characterize the CE stress induced by quinolones, we measured CE stress kinetics of the selected quinolones, the FQ ciprofloxacin, and the NBTI gepotidacin. Rcs stress is activated by various cues and it is unclear which of these results in the observed responses.^21^ Secondary profiling of two other CE stress responses, σE and Cpx, as well as heat shock responses (groES), can provide further insight into the CE process that is affected.^19^ For example, LPS-targeting compounds such as PMBN induce a rapid (<90 min) increase in Rcs and σE stress, while compounds acting on the BAM complex provoke a slower response. Antibiotic compounds that act on intracellular targets typically elicit heat shock (groES) stress.^19^ Thus, we recorded extended stress response profiles of the quinolones in Rcs, σE, Cpx, and heat shock reporter systems.

Stress was measured in real-time during 10 h following compound treatment by normalizing the fluorescence intensity for growth as indicated by the optical density at 600 nm (OD_600_) of the culture. The CluA and CluB compounds, ciprofloxacin, and gepotidacin induced all tested stress systems. An overview of the stress responses for compounds **A2** and **B1**- representative of the responses observed for all compounds in the respective cluster- ciprofloxacin, and gepotidacin at EC80 concentrations (see Table S2) is shown in Figure 3. CluA compounds and ciprofloxacin exhibited similar Rcs kinetics, whereas CluB compounds induced similar Rcs stress kinetics to gepotidacin but were distinct from those of the CluA compounds (Figures S1 and S2). Notably, CluB compounds and gepotidacin induced up to ∼17-fold increase in Rcs stress with a biphasic response pattern, reaching its first peak around 260–280 min post-treatment. In contrast, the CluA compounds and ciprofloxacin induced a single peak around 180–200 min post-treatment, reaching up to a ∼7-fold increase in Rcs stress.

**Figure 3:**
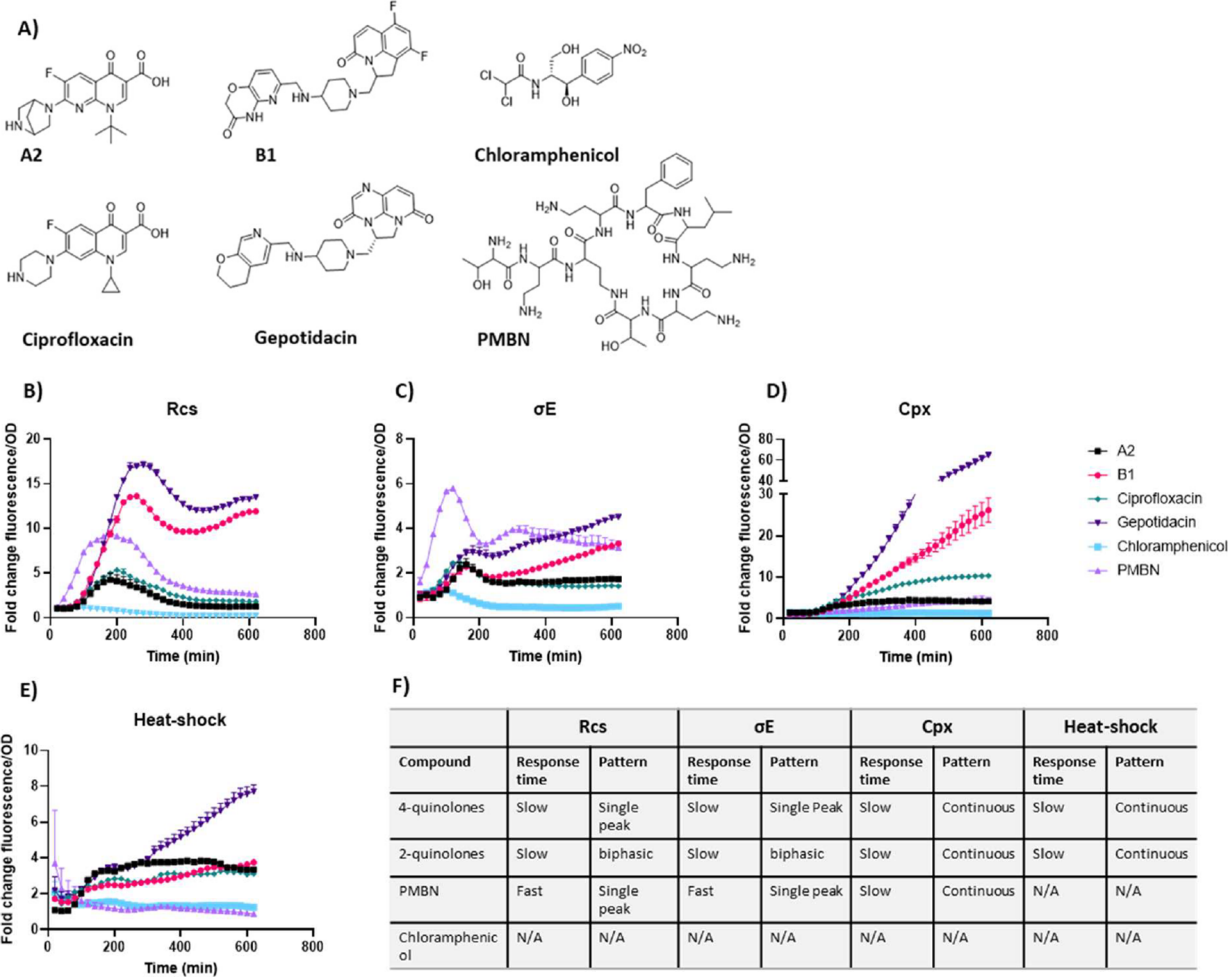
Bacterial stress responses of different compound classes as shown in panel (A). (B) Rcs, (C) σ*E,* (D) Cpx, and (E) heat shock. Graphs show fold change in fluorescence normalized for OD_600_ at EC80 compound concentrations over the course of 10h. Fold change values were determined by comparing OD_600‑_normalized fluorescence of compound-treated samples to DMSO treated samples. PMBN is used as a CE stress-inducing control at 25 μg/mL, which is the concentration used as a control during the screening campaign. Chloramphenicol at 1 μg/mL is used as a non-stress-inducing antibiotic control. Error bars indicate standard deviation of the average of triplicate samples from at least two independent experiments. (F) Overview of the different stress responses based on the compound class.

When considering other stress responses, the two classes of quinolones also consistently show similar kinetics within their cluster, but are distinct from the other cluster. CluB compounds and gepotidacin induced a biphasic σE response. Its primary peak precedes that of the Rcs response (∼150 min compared to 260 min), reaching up to a moderate ∼3-fold change. Following a short drop, the stress then continuously increases throughout the treatment. The Cpx response had a later onset (∼150 to 200 min; around the time of the primary peak of σE stress) compared to σE and Rcs (∼60 and 100 min onset time, respectively) and continuously increased throughout treatment. Heat-shock stress was also rapidly induced and continued to increase throughout the treatment.

CluA compounds and ciprofloxacin also induced a σE response, with a single peak preceding the peak of the Rcs response, reaching up to a moderate ∼3-fold change. Cpx stress was induced early (<100 min) and remained elevated with up to a ∼10-fold increase compared to DMSO-treated cells. Similarly, heat-shock stress was rapidly (<100 min) induced and reached levels up to ∼4-fold higher than control cells. In contrast to the quinolones, PMBN induced a more rapid induction of Rcs and σE stresses than either quinolone cluster and did not induce heat shock. Chloramphenicol, a bacteriostatic compound that inhibits protein synthesis, was used as a negative control^38^ and did not induce any of the stress response systems tested.

Overall, the CE stress profiling revealed activation of all three tested CE stress systems, although it remains unclear which CE process­(es) the quinolones interfere with, and whether the CE stress is a direct effect on the membrane or an indirect consequence of a cytosolic process being inhibited.

### Compounds A2 and B1 Induce Cell Envelope Stress through Gyrase Inhibition

FQ antibiotics and aminopiperidine-linked quinolone NBTIs such as gepotidacin primarily target the cytosolic enzymes gyrase and topoisomerase IV. The two distinct binding sites of both compound classes are well described.^36,39,40^ The slower CE stress kinetics compared to PMBN suggest that the CE stress induced by quinolones could be a secondary effect following inhibition of their primary targets, as opposed to being caused directly, e.g. by membrane insertion or during diffusion of the compound through the CE.^41^

To investigate if gyrase/topoisomerase IV are indeed the primary targets of the CluA and CluB quinolones, we raised resistant mutants to the standard-of-care FQ lomefloxacin (FQ_R_) or the NBTI gepotidacin (GEP_R_), due to limited availability of the hit compounds. Cross-resistance of such resistant strains to the CluA or CluB compounds would indicate that these compounds occupy similar binding pockets to FQs or gepotidacin, respectively, validating them as gyrase inhibitors.

Serial passaging of *E. coli* MC4100 in the presence of increasing concentrations of lomefloxacin or gepotidacin yielded resistant variants of *E. coli* MC4100. We used MC4100 to raise resistance, because unlike the screening strain TOP10F’, this strain has a functional SOS stress response, which has been implicated in developing resistance against quinolones.^42^ Compounds also elicited a robust Rcs stress response in *E. coli* MC4100, allowing for this strain to be used for stress assays.

Sequencing of the *gyrA* gene of an FQ_R_ colony grown in the presence of 20 μM lomefloxacin (50x MIC) revealed that it encoded an S83L substitution in GyrA. This mutation has been previously described to provide FQ resistance in spontaneously resistant laboratory strains as well as clinical isolates.^43,44^ FQ_R_ cells showed cross-resistance to the CluA compound **A2** (Figure S3). They also lacked Rcs stress following lomefloxacin or **A2** treatment (Figure 4). In contrast, the FQ_R_ strains remained susceptible to growth inhibition by the CluB compound **B1** and gepotidacin and still exhibited Rcs stress (Figure S3 and Figure 4 *B and D*). Sequencing of a GEP_R_ colony grown in the presence of 24 μM (3x MIC) did not show any mutation in *gyrA* or *parC*. Instead, point mutations were found in *acrR, marR,* and *rpoC*. Mutations in *acrR* and *marR* indicate changes to multidrug efflux pump expression, and a mutation in *rpoC* may affect transcription initiation by the RNA polymerase which may be a nonspecific resistance mechanism to antibiotic pressure.^45–47^ Mutations in these genes have been previously reported in strains resistant to gepotidacin and indicate a distinct resistance profile of FQ-resistant and gepotidacin-resistant mutants.^48^ GEP_R_ cells showed moderate insensitivity to EC80 levels of gepotidacin and exhibited cross-resistance to the CluB compound **B1** (Figure S3). In addition, GEP_R_ did not show Rcs stress when treated with gepotidacin or **B1**. GEP_R_ mutants were also less susceptible to lomefloxacin and **A2**, and showed similar Rcs stress patterns as observed in FQ_R_ following treatment with these compounds (Figure 4).

**Figure 4:**
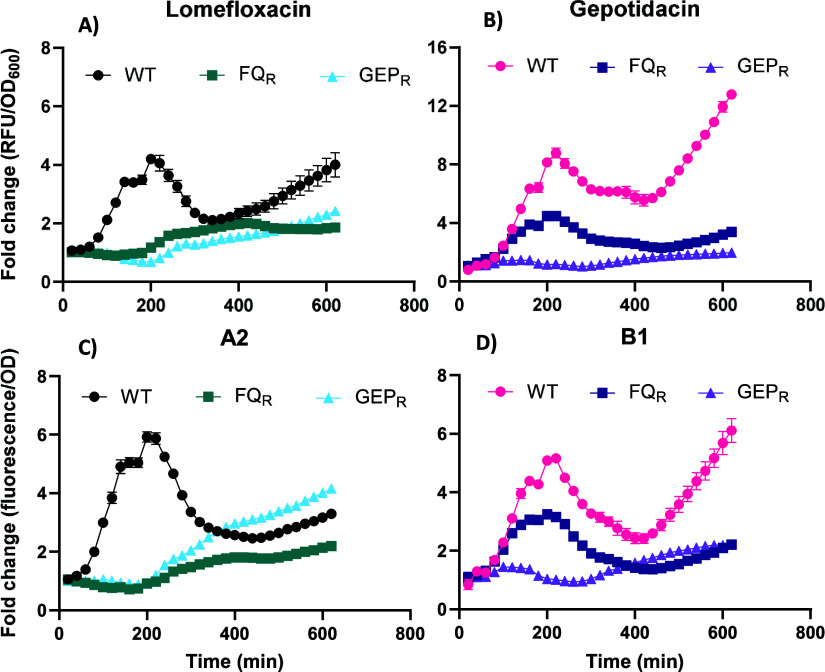
Rcs stress induced by EC80 concentrations of (A) lomefloxacin, (B) gepotidacin, (C) **A2**, and (D) **B1** in wildtype (WT), fluoroquinolone resistant (FQ_R_) or gepotidacin resistant (GEP_R_) bacteria. Graphs show the fold change in fluorescence adjusted for bacterial growth as determined by OD_600_. Error bars indicate the standard deviation of triplicate samples. Data are representative of three independent experiments with similar results.

Together, this suggests that the CluA compounds act on similar binding pockets as FQs and that CluB compounds have a mode of action similar to gepotidacin, distinct from that of FQs. GEP_R_ mutants are somewhat cross-resistant to FQs and the CluA compound **A2**. Cross-resistance to FQ antibiotics is a known consequence of mutations affecting multidrug-efflux pumps. The decrease in Rcs stress following FQ or **A2** treatment is likely a consequence of increased efflux, and thus decreased compound concentration interacting with gyrase. In addition, these data suggest that the observed Rcs stress induction is most likely an indirect consequence of inhibiting gyrase, the primary target of quinolones, rather than arising from a separate mechanism of action.

### Rcs Stress Is Likely Mediated by a Downstream Process Independent of DNA Cleavage and SOS Stress Induction

To further study how gyrase inhibition may cause CE stress, we investigated whether DNA breakage and SOS stress induction could be linked to CE stress. FQ antibiotics and NBTIs like gepotidacin act by simultaneously binding to the gyrase and the DNA within the cleavage complex, causing replication arrest and the formation of double or single-strand DNA breaks, respectively.^36,39,40^ Consequently, DNA breaks trigger the activation of the SOS stress response by derepressing LexA to initiate DNA repair mechanisms.^42^

To examine the potential link between DNA gyrase inhibition, SOS stress, and CE stress, we designed a fluorescent SOS reporter plasmid, similar to the reporter plasmids used to study CE stresses. The plasmid features the gene encoding mNeonGreen under the control of the P_*lexA*_ promoter, showing high fluorescence following treatment of *E. coli* MC4100 with the known SOS stress-inducing compounds lomefloxacin or mitomycin C (Figure S4). Importantly, these compounds did not induce high fluorescence in TOP10F’ carrying the SOS stress reporter plasmid, confirming the absence of a functioning SOS response in this strain.

Both representative hit compounds **A2**and **B1** induced SOS stress at similar levels as the FQ lomefloxacin at EC80 compound concentrations, confirming the presence of DNA damage (Figure 5). In contrast, LEI-800, a novel gyrase inhibitor that has been shown not to cause DNA breaks, did not induce strong fluorescence, indicative of an absence of SOS stress.^49^

**Figure 5:**
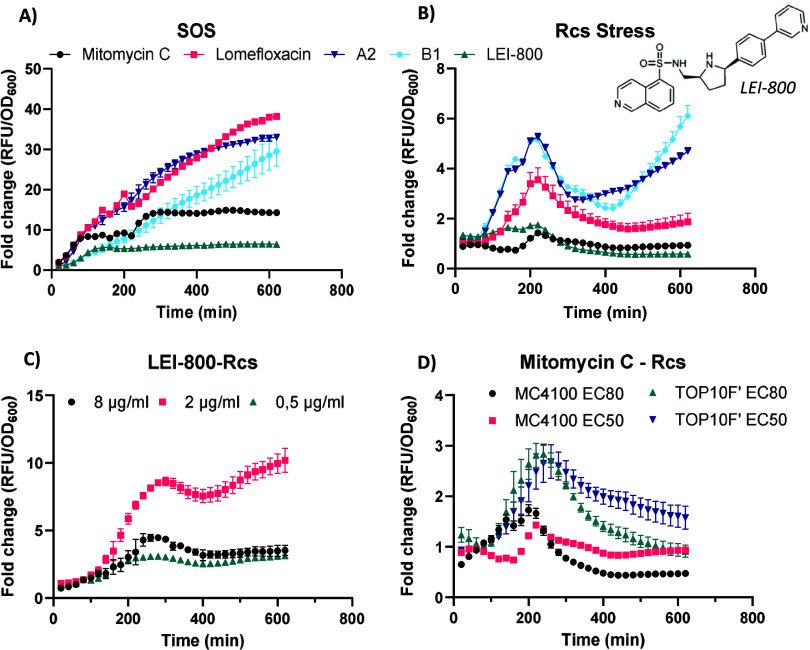
(A) SOS stress induced by different compounds at EC80 concentrations in *E. coli* MC4100. (B) Rcs stress of different compounds at EC80 concentrations in *E. coli* MC4100. (C) Rcs stress of the nonquinolone gyrase inhibitor LEI-800 in TOP10F’. (D) Rcs stress induced by mitomycin C in *E. coli* TOP10F’ and *E. coli* MC4100. Graphs show fold change in fluorescence adjusted for bacterial growth (OD_600_). Error bars indicate standard deviation of triplicates. Data are representative of two independent experiments with similar results.

To further correlate CE and SOS stress, we measured Rcs stress following treatment with LEI-800 and mitomycin C. The latter is a DNA alkylating agent, which induces DNA breaks without interfering with gyrase functioning.^50^ Neither compound induced Rcs stress in *E. coli* MC4100, indicating that neither gyrase inhibition nor DNA breaks alone can induce an Rcs stress response in an SOS-proficient strain. Intriguingly, LEI-800 induced high levels of Rcs stress similar to CluB compounds in the SOS-deficient strain *E. coli* TOP10F’ (Figure 5C). Mitomycin C also induced Rcs stress in TOP10F’, but at lower levels than the gyrase inhibitors (Figure 5D). This suggests that gyrase inhibition alone leads to high levels of Rcs stress in the absence of a functioning SOS stress response. The lower levels of Rcs stress induced by mitomycin C suggest that the DNA breaks that are a consequence of gyrase inhibition may be involved in CE stress to some extent. Still, high levels of Rcs stress are partially a result of another downstream effect of gyrase inhibition.

Taken together, it appears that the SOS stress response is not required to induce CE stress. Instead, it may prevent CE stress following gyrase inhibition. Overall, this suggests that a distinct downstream response secondary to gyrase inhibition and independent of SOS contributes most to the observed CE stress.

### Quinolones Do Not Increase Membrane Permeability

CE stress profiling of the quinolone compounds indicates that while the primary target of gyrase inhibitors is cytosolic, there may be changes to the structure and integrity of the CE. To investigate the effect of our quinolone hits on membrane permeability, we performed a 1-N- phenylnaphthylamine NPN uptake assay (Figure 6), as described previously.^51^ NPN is a hydrophobic fluorophore that is unable to cross the OM under normal conditions. Upon permeabilization of the OM, the dye can interact with the phospholipid bilayer and exhibits increased fluorescence intensity.

**Figure 6:**
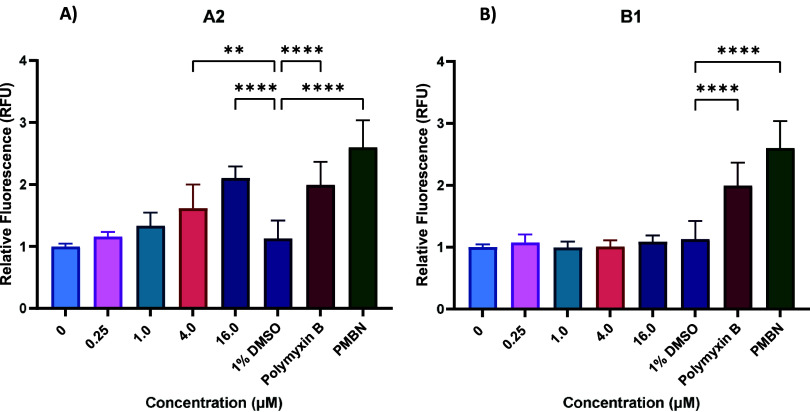
Effect of compounds (A) **A2** and (B) **B1** on membrane integrity analyzed by NPN permeability. Polymyxin B was used at 1 μM and PMBN at 4 μM as membrane permeabilizing control compounds. Data represent the average relative fluorescence values of three independent experiments performed in triplicate. Error bars represent standard deviation of three independent experiments. Data were analyzed using a One-Way ANOVA with Dunnett’s multiple comparisons. **: *p* = 0.0017; ****: *p* < 0.0001.

At the tested concentrations, only **A2** induced a significant increase in relative fluorescence at 4 and 16 μM. However, the EC80 concentration of compound **A2** is around 0.35 μM, indicating that the increased fluorescence values may occur following cell death rather than reflecting increased membrane permeability prior to that. Compound **B1** did not exhibit an increase in fluorescence at the highest tested concentrations. Thus, it appears the quinolones cause CE stress without directly compromising the integrity of the OM.

### Gyrase Inhibitors Affect Membrane Structure

While CE integrity was not compromised by the quinolones, we hypothesized that there likely are visible changes to the CE structure. Therefore, we used phase contrast and fluorescence microscopy to analyze cell morphology. The fluorescent lipid dye Nile Red was used to visualize potential disturbances in bacterial membranes, as previously described.^52^ Hoechst dye was used to stain DNA.

*E. coli* TOP10F’ cells were treated with DMSO or compounds at EC50 concentrations, incubated at 37 °C, and imaged after 4h of treatment (Figure 7). DMSO-treated control cells had normal rod-shaped morphology. Drastic changes to cell and membrane morphology were observed following compound treatment. Both compounds **A2**and **B1** significantly increased the overall average length of the cells in the population and induced extreme elongation in a subset of the cells (Figure 7). A similar trend was observed for the average cell area, indicating that both compounds significantly change cell morphology. Interestingly, FQs are known to induce filamentation of bacterial cells due to activation of the SOS stress response.^53^ However, TOP10F’ cells lack the SOS response, indicating that quinolones can induce filamentation in the absence of a functional SOS stress response.

**Figure 7:**
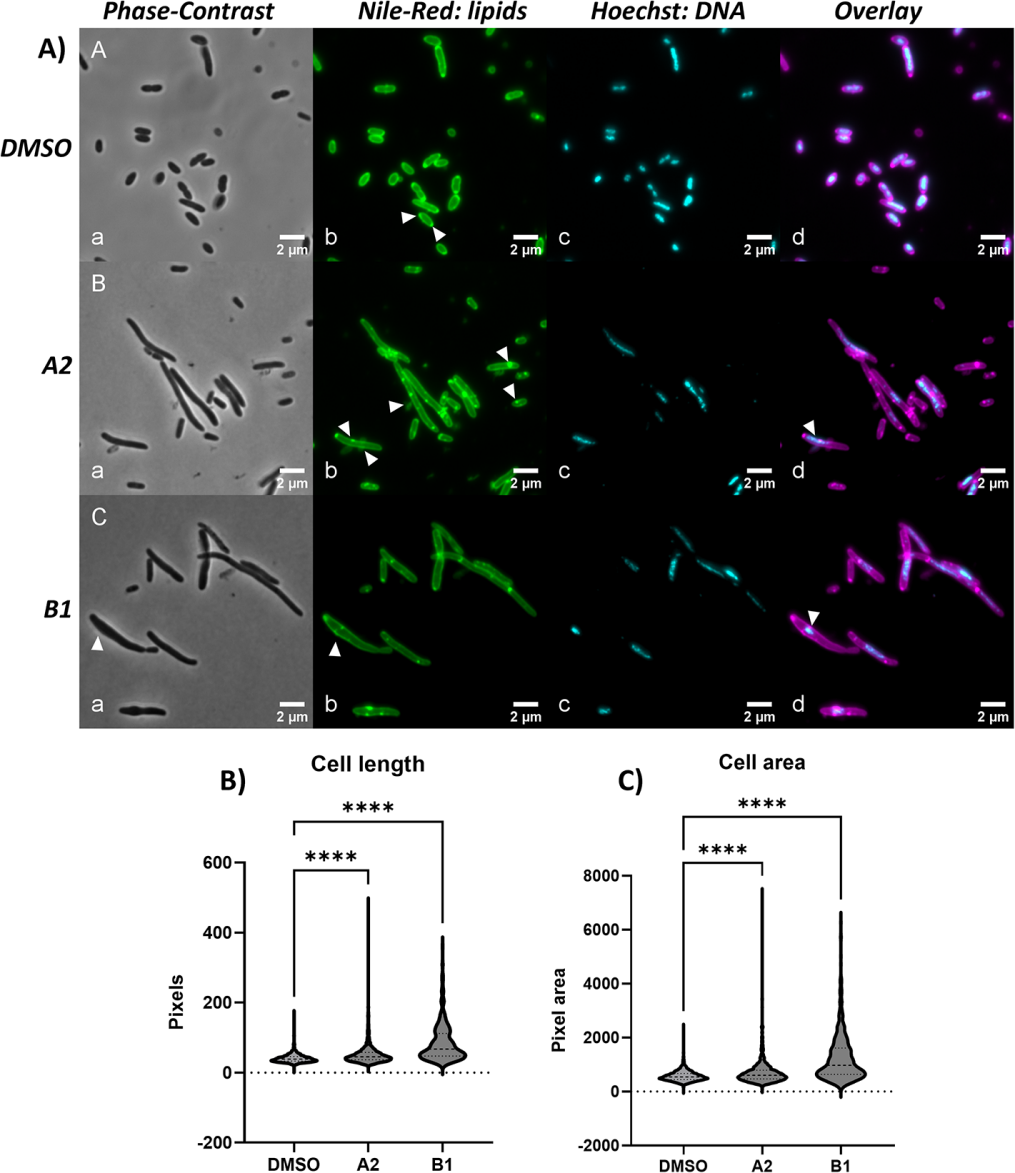
(A) Microscopy images of *E. coli* TOP10F’ treated with DMSO (A) or EC50 concentrations of compounds **A2** (B) or **B1** (C) during 4h (100× magnification). Cells were stained with Nile red to visualize membranes and Hoechst to label DNA. a: Phase Contrast; b: Nile Red; c: Hoechst; d: overlay of Nile Red and Hoechst. White arrows indicate differences observed between control and compound treated cells, such as different localization and increased number of bright, lipid spots, vesicle-like bulges, apparent leakage of lipid contents and condensed DNA. Pictures are representative of at least 5 images taken per sample, during two independent experiments with similar results. Bottom panels show (B) cell length or (C) cell area after treatment with DMSO or EC50 concentrations of **A2** or **B1**. Cells were analyzed after 4h of compound treatment. Data are representative of two independent experiments with similar results. Data were analyzed using a One-Way ANOVA with Kruskal–Wallis test for multiple comparisons. **** = *p* < 0.0001. Cells were counted from 3 to 5 pictures of each condition (DMSO: *n* = 1773, A2: *n* = 1920, B1: *n* = 555). Data are representative of two individual experiments with similar results.

In addition to elongation, cells also had visible vesicle-like protrusions, appeared to leak cell content, and showed enlarged bulges around areas where DNA seemed to condense. Overall, DNA distribution seemed irregular in compound-treated cells, which is likely a consequence of stalled DNA replication due to gyrase inhibition. Strikingly, compound-treated cells also showed changes in lipid distribution. In untreated cells, fluorescence appeared mainly as an even circumferential fluorescence, likely reflecting the cell membranes. More focused bright dots were localized at one or both poles of the cell or the division septa. However, in cultures treated with quinolones, the fluorescent staining was highly irregular, showing an uneven spread of brighter patches along the membrane, and may also have accumulated intracellularly, as previously described.^54^

Next, we examined the surface of compound-treated cells in more detail using scanning electron microscopy (SEM) imaging (Figure 8). Indeed, cells treated with compounds **A2** and **B1** showed drastically altered surfaces. Overall, compound-treated cells exhibited similar changes to those observed with phase-contrast microscopy: elongation, extensive surface damage with leakage of intracellular components, and the formation of vesicle-like protrusions, which may indicate the onset of cell lysis. Interestingly, these protrusions have a very smooth appearance, unlike the surrounding cell surface. They possibly represent inner membrane material that bulges through the compromised peptidoglycan/outer membrane layer. Additionally, as suggested by fluorescence membrane staining, there were clear changes to the cell surface structure. DMSO-treated control bacteria appeared as intact, rod-shaped cells with no visible cell wall rupture or collapse and a relatively rough surface. In contrast, the surface of compound-treated cells appeared “stretched out” and smooth, featuring irregular wrinkles and deeper groove-like rifts.

**Figure 8:**
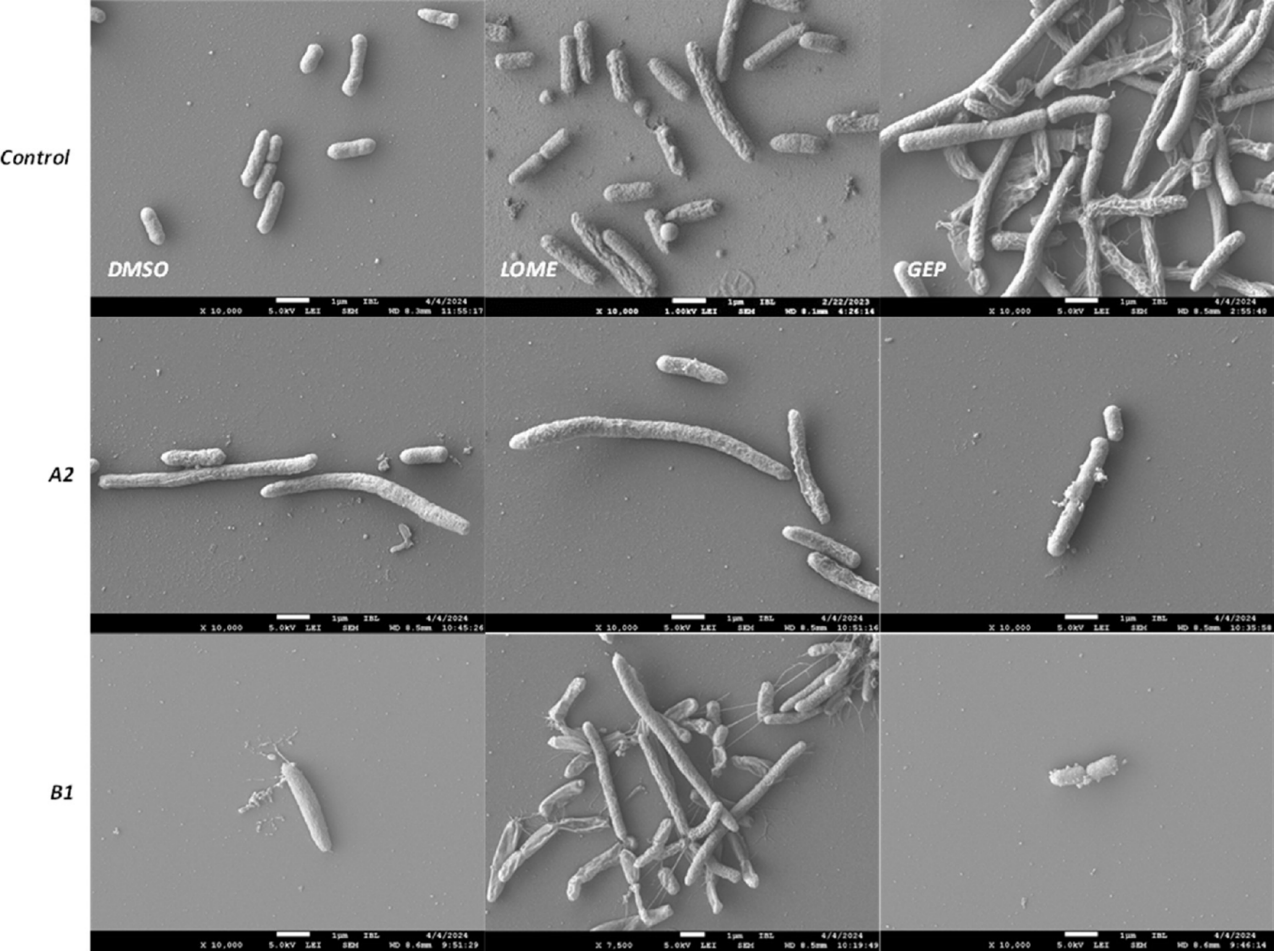
SEM images of *E. coli* TOP10F’ cells treated with DMSO or EC50 concentrations of lomefloxacin (LOME), gepotidacin (GEP) or compounds **A2** or **B1** for 4h. Pictures shown are all at 10.000 × magnification, except the middle picture of B1, which is at 7.500 × magnification. Pictures are representative of at least 5 images taken per sample, during 2 independent experiments with similar observations.

Overall, the cell morphology of cells treated with our hit compounds **A2** or **B1** appeared substantially altered, indicating that gyrase inhibition leads to profound changes in the CE. The measured CE stress likely correlates with these changes.

## Discussion

In this study, we performed a fluorescence-based high-throughput screen to identify small molecules that induce Rcs stress, potentially by interfering with CE integrity. This screen was the first instance at the ELF where a phenotypic bacterial assay was successfully adapted to a 1536-well format. The assay proved highly robust in this format and allowed for the identification of antibiotic compounds. Interestingly, the final hit list consisted predominantly of quinolones, which are not generally recognized for membrane-disrupting properties. Instead, quinolones are known and well-studied pharmacophores for compounds targeting bacterial gyrase/topoisomerase IV.^35,36^ Decades of experience with clinical antibiotics targeting the bacterial DNA gyrase have established this enzyme as an excellent antibacterial target because its activity is essential for the cyclic processes of DNA replication and transcription, which can be interrupted at multiple stages by addressing a variety of target sites.^55^

FQs represent one of the most widely used antibiotic classes. They are active against a broad range of bacterial species, including the difficult-to-treat **Mycobacterium tuberculosis*.* ^27,56^ However, FQs face increasing resistance rates, warranting urgent action to find compounds active against FQ-resistant strains.^27^ Several compounds targeting gyrase with mechanisms of action that differ from that of FQs, the so-called NBTIs, are being evaluated in the clinic.^57,58^

Our screen successfully identified two types of gyrase inhibitors that did not show toxicity against mammalian cells: CluA compounds similar to FQs and CluB compounds similar to aminopiperidine-linked quinolones belonging to a class of NBTIs. The latter make up a promising category of compounds currently being clinically evaluated as antibiotics–with the recently FDA-approved gepotidacin being the most prominent example.^29,30^ This highlights that novel types of gyrase inhibitors are a highly promising class of antibiotics in an era where novel classes of antibiotics are scarce. Importantly, NBTIs, including compound **B1** identified here, are active against FQ-resistant strains and are thus promising candidates to treat infections caused by resistant bacteria.^59^

Despite identifying antibiotic compounds, the question arises as to why no “true” membrane-acting compounds were picked up in the Rcs screen. There are several reasons why the outcome of the screen may have been skewed toward identifying compounds such as quinolones, including the composition of the ELF library, the screening and selection procedure, and the nature of compounds that typically target the CE. First, the exact contents of the ELF library are unknown to the users but comprise exclusively small molecules designed to have drug-like properties. However, many antibiotics with targets in the CE are complex natural-product-derived compounds or peptidomimetics, not present in this type of library.^60^ Instead, screening a natural product library using the Rcs reporter assay may yield candidates that more directly target the CE and its biogenesis.

Second, during the selection process, molecules were triaged based on their drug-like properties, potentially discarding less suitable but nonetheless potent compounds that, with SAR studies and chemical optimization, may be turned into drug candidates. Finally, compounds were screened at a single concentration for Rcs stress-induction without assessing bacterial growth. Hence, growth inhibition was not used as a selection criterion. This could have resulted in missing out on compounds that may induce a rapid stress response while resulting in cell death. Nevertheless, the library’s composition has the advantage that hits are already prioritized for drug-like properties and could be more rapidly optimized for clinical use.

The rather unexpected outcome and the serendipitous discovery of mostly quinolones as Rcs stress inducers led us to pursue the source of CE stress induced by these gyrase inhibitors. The effects of sublethal concentrations of gyrase inhibitors on the bacterial CE have been sparsely described in the literature, and the exact mechanism remains unknown. Quinolones were previously found to induce LPS release, change cell-surface hydrophobicity, and increase permeability in **E. coli*.* ^61,62^ One recent study has shown that cells treated with quinolones undergo membrane remodeling, especially involving alterations to lipid composition.^63^ Transcriptomics of *E. coli* treated with the FQ ofloxacin has shown significant changes in expression of OmpF genes involved in peptidoglycan synthesis and LPS biosynthesis, indicating significant changes to the CE.^64^ Of note, novobiocin, a coumarin antibiotic that targets the ATPase unit of GyrB, has been shown to accelerate LPS transport, alter CE permeability and synergize with polymyxin B.^65,66^ However, this effect can be directly attributed to a separate mechanism of action through a direct interaction with the LPS transporter subunit LptB, independent of gyrase inhibition. In contrast, we show that gyrase inhibition directly induces CE perturbance.

We provide evidence that the CE stress observed during quinolone treatment is likely a secondary effect following gyrase inhibition. The two distinct clusters of quinolones picked up in the screen resulted in different stress patterns, which may be related to their different mechanisms of gyrase inhibition. Both compound classes bind simultaneously to the gyrase and the DNA within the cleavage complex, causing interference with DNA replication, albeit through different binding mechanisms. FQs and likely the CluA compounds trap the DNA in the cleavage complex and inhibit the religation of double-strand breaks, arresting the supercoiling process.^35^ The quinolone binding pocket is well-defined, and the mutation found at amino acid residue S83 in our FQ_R_ mutant is an important interaction site with established FQ antibiotics. Cross-resistance against compound **A2** indicates that it likely targets the same binding pocket. In contrast, aminopiperidine-linked NBTIs such as gepotidacin, and likely also the CluB compounds, stabilize single-stranded cleavage complexes and do not induce the formation of double-strand breaks.^39^ ,^67^ Our GEP_R_ mutant did not show mutations in gyrase, but in the regulator of the AcrAB efflux pump. Gepotidacin has been described to induce mutations in genes affecting efflux pump expression before gyrase mutations arise.^48^ Compound **B1** and gepotidacin both showed similar patterns GEP_R_ mutants and remained active in FQ_R_ mutants. While this does not directly confirm gyrase as a target for **B1**, based on similarities in activity, resistance profiles, and structure between **B1** and the confirmed gyrase-targeting gepotidacin, we suggest that **B1** acts on a similar target site as gepotidacin.

FQs and consequent double-stranded DNA breaks are known inducers of the SOS stress response.^68,69^ In some cases, FQs and subsequent activation of SOS stress have been implicated in changes to bacterial morphology and CE structure, including filamentation, membrane depolarization, and changes in the protein/lipid ratio.^62,70–72^

To our knowledge, this is the first study that reveals a relationship between gyrase inhibition and CE stresses such as Rcs and σE. Using SEM, we show that gyrase inhibition by quinolones leads to extensive changes in bacterial surface appearance, which likely contributes to the measured CE stress. The vesicle-like protrusions could be OM vesicles or inner membrane protruding through gaps in the OM. Intriguingly, cell elongation is often associated with the activation of the SOS stress response.^42^ However, *E. coli* TOP10F’ cells, which are unable to induce SOS stress, still formed filaments when treated with gyrase inhibitors and induced higher levels of CE stress than the SOS-proficient strain *E. coli* MC4100. This points toward a potentially distinct, SOS-independent driver of cell elongation. Notably, ofloxacin has been found to induce other stress responses, including phage shock, and downregulate genes related to cell division, which may be involved in cell elongation.^64^

While our data may suggest a protective role of the SOS response against gyrase-induced CE stress, findings of the nonquinolone gyrase inhibitor LEI-800 suggest an entirely SOS-independent mechanism. LEI-800 does not create double-strand breaks and only induced low levels of SOS stress and no Rcs stress in MC4100, but still activated high levels of Rcs stress in *E. coli* TOP10F’.^49^ This is inconsistent with the hypothesis that SOS stress has a protective role against CE stress induced by gyrase inhibition. Instead, it suggests a strain-dependent response to gyrase inhibition independent of SOS stress. Furthermore, we showed that the DNA intercalating agent mitomycin C does not induce high levels of Rcs stress in MC4100 and TOP10F’, despite inducing high levels of SOS stress in MC4100. Importantly, mitomycin C does cause double-strand DNA breaks but not through gyrase inhibition.^50^

## Conclusions

Combined, the data point toward a still unknown mechanism that is not solely dependent on DNA damage and SOS stress as the main driver of CE stress following gyrase inhibition. Additionally, these findings indicate that the Rcs stress reporter assay can identify gyrase inhibitors and potentially other compounds targeting cellular processes that indirectly affect the CE, especially in SOS-deficient strains. While this highlights the utility of CE stress-based assays as tools for identifying new antimicrobial agents, it also raises the need for further optimization of the assay or library composition to identify more specific CE-targeting compounds.

## Materials and Methods

### Bacterial Strains and Plasmids

Bacterial strains and sources are provided in Table 3. Plasmids and their sources are listed in Table 4.

**Table 3:** Bacterial Strains

species	strain name	comment	origin
*E. coli*	TOP10F′	Wild-type	Thermo Fisher Scientific
*E. coli*	MC4100	Wild-type	^19^
*E. coli*	fluoroquinolone resistant (FQ_R_)	MC4100 with gyrA S83L mutation	This study
*E. coli*	gepotidacin resistant (GEP_R_)	MC4100 with acrR Y49H, marR frame shift at T124 and rpoCI1357S mutations	This study

**Table 4:** Plasmids

plasmid name	reported stress	ref
PuA66_rprA_mNG	Rcs	^20^
PuA66_rpoE_mNG	σE	^20^
PuA66_cpxP_mNG	Cpx	^20^
PuA66_groES_mNG	heat-shock	^20^
PlexA_mNG	SOS	this study

### Chemicals and Compounds

All chemicals were ordered from Merck KGaA, Darmstadt, Germany unless otherwise specified. Compounds used during the initial screen were obtained from the ELF screening library. Hit compounds used throughout the remainder of the study were resynthesized by Symeres (Nijmegen, The Netherlands). At least 10 mg of each compound at ≥95% purity was supplied for retesting. Purity of compounds was assessed using HPLC and ^1^H NMR. MRL-494 of ≥ 95% purity was kindly synthesized and provided by prof. Nathaniel Martin, Leiden University, The Netherlands.^15^ LEI-800 was kindly provided by dr. Mario van der Stelt, Leiden University, The Netherlands. Compound stocks for testing were dissolved in 100% DMSO.

### Growth Conditions

Bacteria were grown in M9 minimal medium (6 g/L Na_2_HPO_4_, 3 g/L KH_2_PO_4_, 0.5 g/L NaCl, 1 g/L NH_4_Cl, 0.4% glucose, 0.1% casamino acids, 1 mM MgSO_4_, 0.1 mM CaCl_2_, 0.0001% thiamine, pH 7.4), supplemented with 1% Lysogeny Broth (LB) for all assays unless otherwise indicated. Antibiotics were supplemented as needed for plasmid maintenance. Liquid cultures were grown at 37 °C, with shaking at 200 rpm.

### HTS Screening/Stress Assay

Overnight cultures (ONCs) were prepared by inoculating 5 mL medium from glycerol stocks and incubating the culture for 16–18 h. ONCs were then diluted to an OD_600_ of 0.05 and grown until an OD_600_ of 0.3–0.4 was reached.

Assay robustness was confirmed by testing its sensitivity to assay-interfering agents, including colored, aggregating and fluorescent compounds. The assay proved largely insensitive to interference. For HTS, bacteria were diluted 50x in prewarmed medium. Fifteen nL of assay compounds (10 μM), DMSO (0.25%), or the reference compound polymyxin B nonapeptide (PMBN) at 25 μM were added to 1536 well plates. Next, 6 μL culture aliquots were added to assay plates containing compounds. Plates were briefly centrifuged for 1 min at 150 x *g*. Plates were incubated at 37 °C with high humidity and fluorescence (Ex./Em. λ: 485/528 nm) was measured at *T* = 0 and *T* = 150 min using a ClarioStar plate reader (BMG Labtech). For data analysis, *T* = 0 values were subtracted from *T* = 150 values to calculate Z-score and % effect compared to the reference compound.

Dose–response curves of the 340 initial active compounds were determined following the preparation of serial dilutions (20 nM–20 μM; 7 points, √10). The % effect compared to the reference compound PMBN was calculated and plotted for each compound dilution. The resulting plots were used to calculate pEC50 (-log­(EC50), with EC50 being determined as the concentration at which a 50% effect is observed).

For lower throughput analysis under laboratory conditions, cultures grown to OD_600_ of 0.3–0.4 were diluted to a final concnetration of 0.05 OD_600_ units/mL in black, clear-bottom 96-well plates (Greiner Bio-One B.V.). Plates were incubated in a Biotek HTX or H1 plate reader (Agilent Technologies) at 37 °C with continuous shaking. Kinetic studies were performed by measuring OD_600_ and fluorescence (Ex./Em. Λ: 485/528 nm) every 20 min for 10h. For end point measurements, fluorescence was measured at *T* = 0 and once more at *T* = 150 min.

### Deselection (Toxicity) Assay

HepG2 cells (H-8065, ATCC) were maintained at 30–80% confluency in medium (EMEM, ATCC) + 10% fetal bovine serum (FBS) (Life Technologies, 50 U/ml Pen/Strep (Life Technologies) at 37 °C, 5% CO_2_ in a humidified incubator. Cells at 60–80% confluency were harvested with 0.05% trypsin-EDTA (ThermoFisher), pelleted at 250 × *g* for 5 min and resuspended in 10 mL prewarmed medium. 1000 HepG2 cells (200,000 cells/ml) were added to each well in 1536 well plates, supplemented with 10 μM staurosporine (Sigma-Aldrich) and 1% DMSO (Fisher). Plates were centrifuged at 250 × *g* for 1 min and incubated for 72 h at 37 °C, 5% CO_2_ in a humidified incubator. Viability of cells was assessed by adding 1.25 μL Cell titer Glo reagent (Promega) and measuring luminescence immediately using a ClarioStar plate reader (BMG Labtech).

### MIC Determination

The antibacterial activity of the compounds was determined by measuring their minimum inhibitory concentration (MIC) values using the broth microdilution method according to the Clinical and Laboratory Standards Institute guidelines (CLSI).^73^ Briefly, a single colony from blood agar plates was inoculated into cation-adjusted Mueller-Hinton broth (CAMHB) and grown to OD_600_ = 0.5. Next, 10^6^ CFU/mL were added to a 2-fold serial dilution series of test compounds and incubated at 37 °C, 600 rpm overnight (18–20 h for Gram-negative strains, 20–24 h for Gram-positive strains), after which the plates were visually inspected for bacterial growth. MICs are reported as the median of triplicates.

### Cell Permeability/NPN Assay

ONC of *E. coli* TOP10F’ grown in LB were diluted to OD_600_ = 0.1 and subsequently grown until OD_600_ 0.5 was reached. Bacterial cells were then pelleted at 1,000*g* for 10 min and resuspended in assay buffer (5 mM HEPES + 20 mM glucose, pH 7.2) to 1.0 OD_600_ units/ml. The cell suspension was then added to a black, clear bottom 96-well plate (Greiner Bio-One) at a final concentration of 0.5 OD_600_ units/ml. Compounds diluted in assay buffer were added to the plate at the desired final concentration. The fluorescent probe *N*-phenyl-1-naphthylamine (NPN) was added at a final concentration of 10 μM. NPN fluorescence (Ex./Em. λ 355, 420) was measured after 1h incubation at room temperature. Relative fluorescence was calculated following the correction of NPN fluorescence in the absence of compound:

$$\mathrm{NPN}\mathrm{uptake}=[F_{\mathrm{obs}}-\mathrm{Fb}]/[F_{\mathrm{control}}-\mathrm{Fb}]$$

Fb = Background NPN (no bacteria)

*F*_control_ = Bacteria + NPN (no compound)

*F*_obs_ = Bacteria + NPN + compound

### Microscopy

#### Fluorescence Microscopy

Bacteria at an OD_600_ = 0.05 were treated with EC50 concentrations (Table S2) of compounds in a 96-well plate for 4 h at 37 °C, shaken at 200 rpm. Cells were collected at 4600*g* for 5 min. Cell pellets were resuspended in PBS with 0.02 mg/mL Hoechst to a final volume corresponding to 10 OD_600_ units/ml and incubated at room temperature for 15 min. Cells were pelleted and resuspended in PBS with 2.5% formaldehyde to a final volume corresponding to 5 OD_600_ units/ml. For membrane staining, Nile Red was added to fixed cells at a final concentration of 50 μM and incubated at room temperature for 15–30 min. Five μL samples were spotted onto 1% agarose slides and imaged using an Olympus IX83 microscope (Olympus) with an ORCA Flash 4.0 LT camera (Hamamatsu).

Images were processed using ImageJ. Cell length and width were quantified using Oufti.^74^ Image data were analyzed using GraphPad Prism 10.3.1. One-way ANOVA was used to perform statistical analysis.

#### Scanning Electron Microscopy

Bacteria at an OD_600_ = 0.05 were treated with EC50 concentrations (Table S2) of compounds identical to treatment for fluorescence microscopy. Bacteria were grown in 96-well plates for 4h at 37 °C with shaking at 200 rpm. 100 μL of culture from pooled wells was added to coverslip glasses coated with poly-l-lysine. After letting the samples rest for a few min, excess liquid was removed, and the samples were fixed by submerging them in 2% glutaraldehyde for 15 min. Samples were then dehydrated by submerging them in increasing acetone concentrations from 70% until 100%. The samples were critical-point dried using a CPD030 critical point dryer (Baltec) and coated with palladium–platinum using a Q150TS plus sputter coater (Quorum). Imaging was performed using a JSM-7600F scanning electron microscope (Jeol).

### Generation of Resistant Mutants

ONC of *E. coli* MC4100 were diluted to OD_600_ = 0.07 in 20 mL LB supplemented with an initial concentration of 0.4 μM lomefloxacin, 8 μM gepotidacin, or no antibiotic. After overnight incubation, part of the culture was plated onto LB agar plates with the same concentration of lomefloxacin or gepotidacin to isolate resistant colonies. OD_600_ of the remaining culture was determined and diluted to OD_600_ = 0.1 into fresh medium with a 1.5–2-fold increased concentration of lomefloxacin or gepotidacin. This procedure was repeated several times until resistant colonies against 50x MIC lomefloxacin and 3× MIC gepotidacin were isolated. Resistant colonies were sent for Whole Genome Sequencing using short Illumina reads at MicrobesNG (Birmingham, UK). Sequencing data was analyzed using CLC Genomics Workbench (Qiagen).

## Supplementary Material



## Notes

Additional experimental data; Table S1, DRCs of hit compounds; Table S2, EC values of hit compounds; Figure S1, CE and heat shock stress kinetics of CluA compounds; Figure S2, CE and heat shock stress kinetics of CluB compounds; Figure S3, growth kinetics of lomefloxacin, gepotidacin, A2, and B1 on WT, FQ_R_, and GEP_R_ mutants; and Figure S4, SOS stress induced by mitomycin C and lomefloxacin (PDF)

## List of Abbreviations

CE: cell envelope
CluA: Cluster A
CluB: Cluster B
DRC: dose–response curve
ELF: European Lead Factory
FQ: fluoroquinolone
NBTI: novel bacterial topoisomerase inhibitor
OM: outer membrane
OMP: outer membrane protein
PMBN: polymyxin B nonapeptide
QHL: qualified hit list
Rcs: regulator of capsular synthesis
