# Supplementary material for: An Rcs Stress-Based High-Throughput Screen Reveals Novel Gyrase Inhibitors as Indirect Inducers of Cell Envelope Stress in Gram-Negative Bacteria
Source: ACS Infect Dis. 2025 Aug 8;11(9):2577–92. doi: 10.1021/acsinfecdis.5c00445 (PMC12442099; doi:10.1021/acsinfecdis.5c00445)
Supplement: Supplementary file 1 [file id5c00445_si_001.pdf]

An Rcs stress-based high-throughput screen reveals novel gyrase inhibitors as indirect inducers of cell envelope stress in Gram-negative bacteria.

Supporting information

Laurence Cleenewerk[1], Alexandra Otto[1], Willemijn Wouters[2] , Joost Willemse[3], Meiling Gao[4], Vladyslav Lysenko[4], Jeroen M. Punt[5], Mario van der Stelt[5], Nathaniel I. Martin[4], Peter van Ulsen[1], Joen Luirink[1,].*

[1]Department of Molecular Microbiology, A-LIFE, AIMMS, VU Amsterdam, De Boelelaan 1108, 1081 HZ Amsterdam, The Netherlands.

[2]Pivot Park Screening Centre, Kloosterstraat 9, 5349 AB Oss, The Netherlands

[3] Institute of Biology, Microscopy Unit, Leiden University, Sylviusweg 72, 2333 BE Leiden, The Netherlands

[4]Biological Chemistry Group, Institute of Biology, Leiden University, Sylviusweg 72, 2333 BE Leiden, The Netherlands

[5]Department of Molecular Physiology, Leiden Institute of Chemistry, Leiden University, Einsteinweg 55, 2333 CC Leiden, The Netherlands

** Email: s.luirink@vu.nl*

Supplemental information

Table ST1: *Dose-Response curves (DRCs) of the hit compounds during the primary screen and of the resynthesized compounds. A DRC was prepared with concentrations ranging from 20 nM – 20 µM. (7 points, √10, n = 2). The MAX effect of each concentration was calculated and plotted to calculate pEC50. pEC50 is defined as -log of the concentration (mol/l) at which 50% effect is observed.*

**Com-pound**	**DRC (primary screen)**	**DRC (resynthesized)**	**pEC50 (resyn.)**
A1	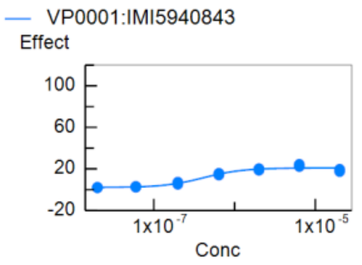	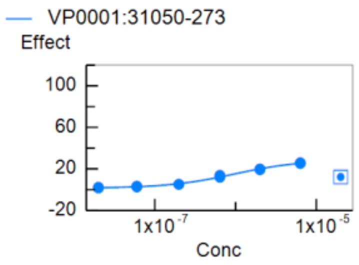	6.02
A2	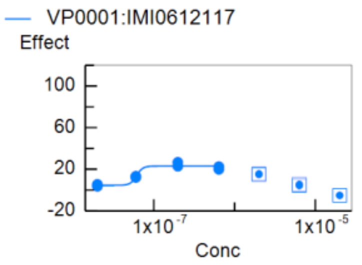	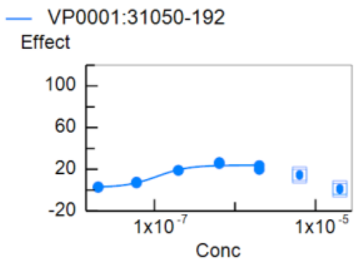	6.97
A3	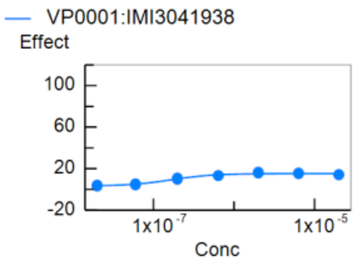	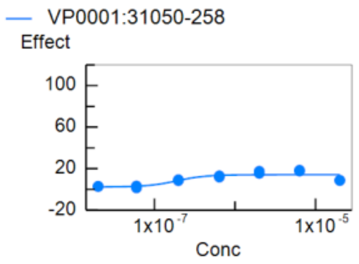	6.72
A4	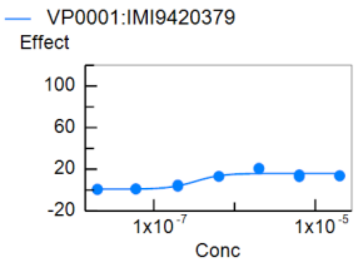	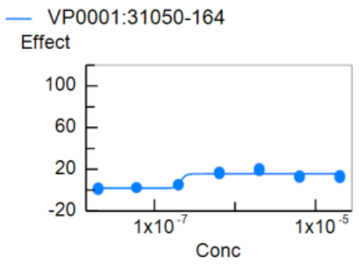	6.67

<table>
<tr><td>B1</td><td>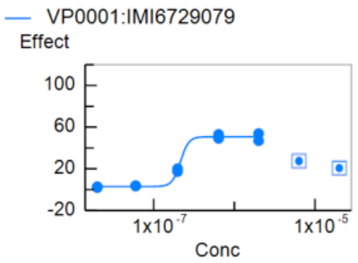</td><td>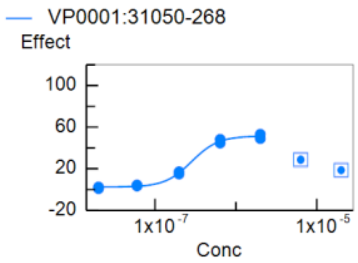</td><td>6.55</td></tr>
<tr><td>B2</td><td>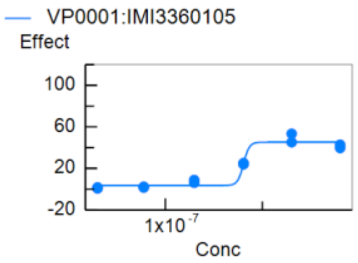</td><td>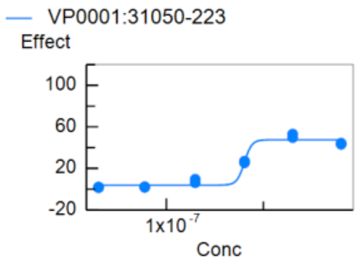</td><td>6.2</td></tr>
<tr><td>B3</td><td>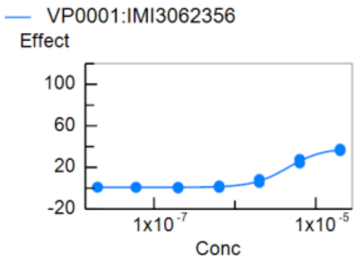</td><td>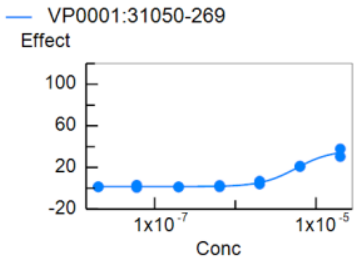</td><td>5.25</td></tr>
<tr><td>S1</td><td>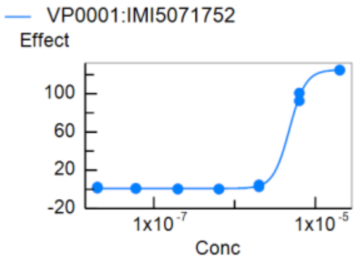</td><td>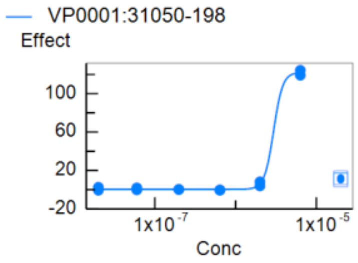</td><td>5.53</td></tr>
<tr><td>S2</td><td>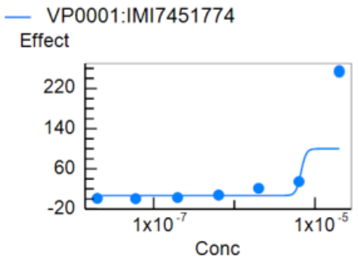</td><td>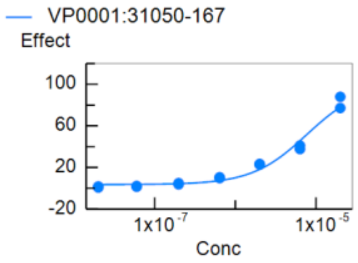</td><td>5.11</td></tr>
</table>

Table ST2: *Concentrations of compounds at which growth after 7 hours is inhibited by 80% (EC80) or 50% (EC50), compared to DMSO control. Concentrations are shown in µg/ml.*

		TOP10F'		MC4100	
	Compound	***EC80***	***EC50***	***EC80***	***EC50***
Cluster A	A1	0,063	0,016	2	0,5
	A2	0,063	0,016	0,25	0,125
	A3	0,031	0,0078	1	0,25
	A4	4	2	1	0,25
Cluster B	B1	0,5	0,031	2	0,5
	B2	2	<0,063	16	2
	B3	1	0,016	>16	2
Other Compounds	MRL-494	32	16	n/d	n/d
	Ciprofloxacin	>0,063	0,002	0,016	0,008
	Lomefloxacin	2	0,031	0,25	0,125
	Gepotidacin	8	0,125	8	1
	LEI-800	8	1	8	2

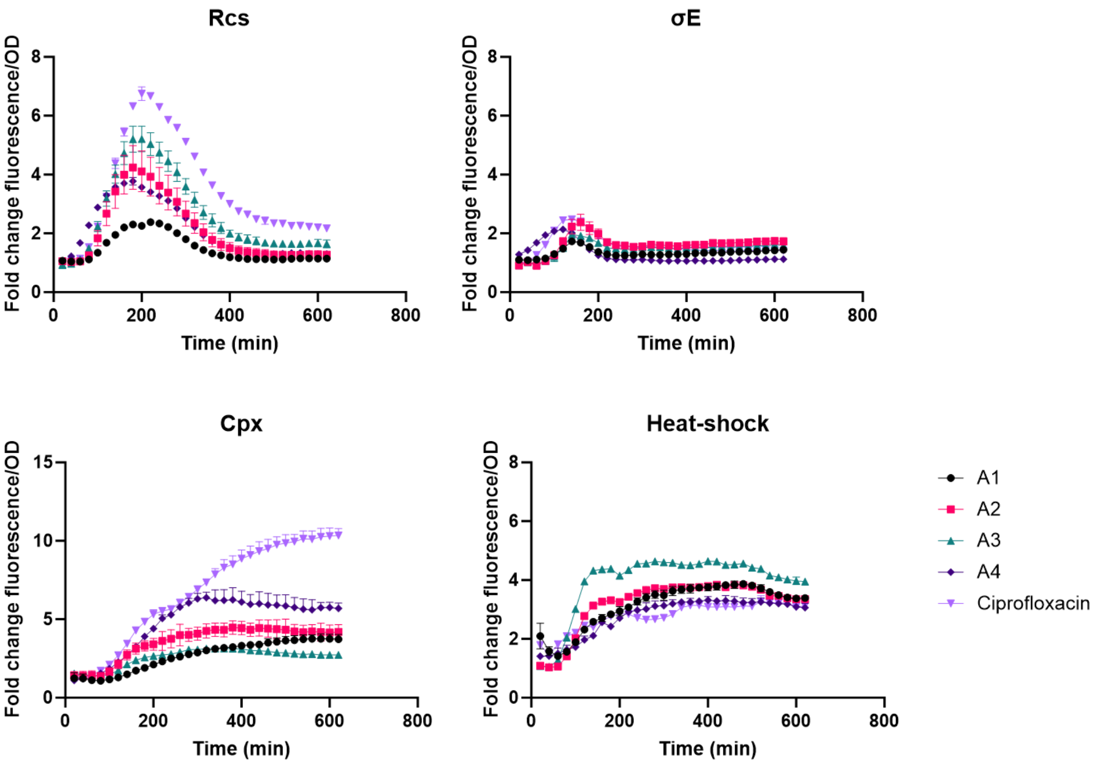

Figure S1: CE and heat-shock stress kinetics of CluA compounds and ciprofloxacin. E. coli TOP10F' cells were treated with EC80 concentrations of compounds and Rcs stress induced fluorescence corrected for growth (OD_{600}) recorded over time. Error bars indicate standard deviations of triplicates. Data are representative of at least two independent experiments.

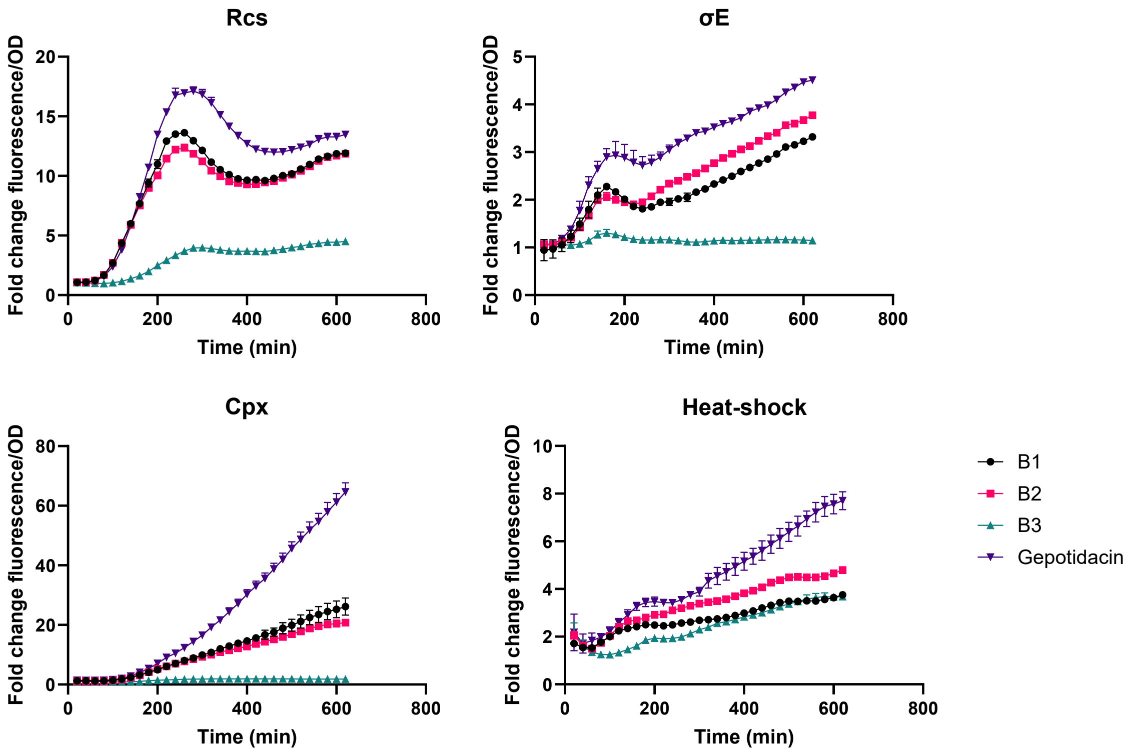

Figure S2: CE and heat-shock stress kinetics of CluB compounds and gepotidacin. E. coli TOP10F' cells were treated with EC80 concentrations of compounds and Rcs stress induced fluorescence corrected for growth (OD_{600}) recorded over time. Error bars indicate standard deviations of triplicates. Data are representative of at least two independent experiments.

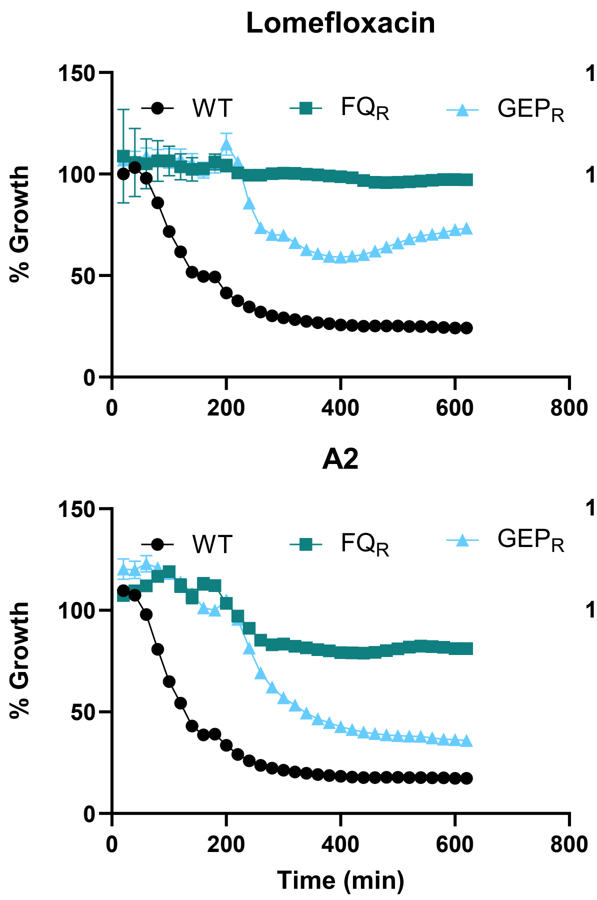

*Figure S3: Growth kinetics of lomefloxacin, gepotidacin, **A2** and **B1** on WT, FQ_R and GEP_R mutants. E. coli TOP10F' cells were treated with EC80 concentrations of compounds and growth (OD_{600}) recorded over time. Graphs indicate % growth compared to DMSO control. Error bars indicate standard deviations of triplicates. Data are representative of three independent experiments with similar results.*

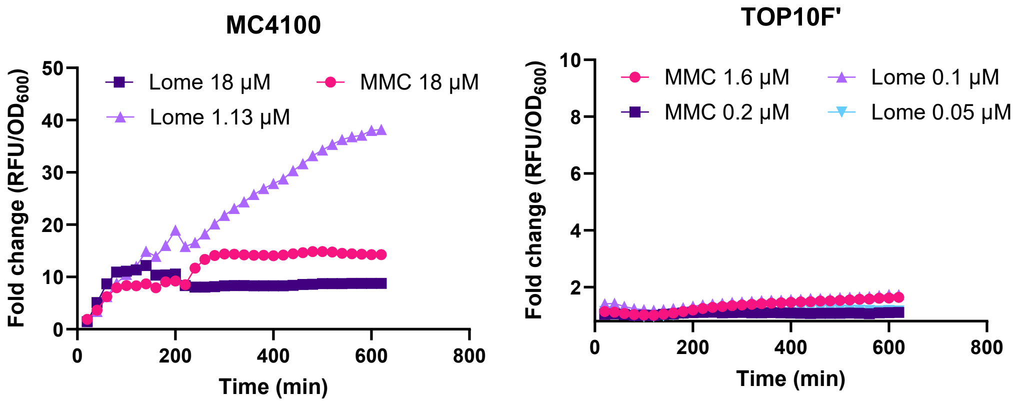

*Figure S4: SOS stress induced by mitomycin C (MMC) and lomefloxacin (Lome). **A)** E. coli MC4100 or **B)** TOP10F' cells with a plasmid expressing mNeonGreen under control of a PlexA promotor were treated with compounds and growth (OD_{600}) and fluorescence recorded over time. Graphs indicate fold change in growth-adjusted fluorescence compared to DMSO control. Error bars indicate standard deviations of triplicates.*
